# High-efficiency radiation beyond the critical angle via phase-gradient antireflection metasurfaces

**DOI:** 10.1515/nanoph-2024-0545

**Published:** 2025-02-03

**Authors:** Xiaoxuan Ma, Hainan He, Runqi Jia, Hongchen Chu, Yun Lai

**Affiliations:** National Laboratory of Solid State Microstructures, School of Physics, Collaborative Innovation Center of Advanced Microstructures, and Jiangsu Physical Science Research Center, 12581 Nanjing University, Nanjing 210093, China; School of Physics and Technology, Nanjing Normal University, Nanjing 210023, China

**Keywords:** high-efficiency radiation, phase-gradient antireflection metasurfaces, total internal reflection

## Abstract

Total internal reflection generally occurs at incident angles beyond the critical angle, confining electromagnetic waves in dielectrics with higher refractive indices. In this work, we present a metasurface-based solution to transform such total reflection into high-efficiency transmission. We demonstrate that a phase-gradient antireflection metasurface designed on the dielectric surface not only compensates for the transverse wave vectors of the incident and transmitted waves but also addresses the impendence mismatch between the two media, eventually achieving high-efficiency transmission with flexibly-controlled wavefronts beyond the critical angle. The design of this unique metasurface is enabled by applying the reciprocity principle to circumvent the traditional limitation of total internal reflection. The theory and functionalities of the phase-gradient antireflection metasurfaces are verified through both simulations and microwave experiments. Our work opens a new avenue for high-efficiency radiation manipulation beyond the critical angle, enabling rich applications such as high-efficiency waveguide-to-free-space couplers, high-radiation-efficiency quantum dots, and high-radiation-efficiency light-emitting diodes.

## 1 Introduction

**Figure 1:**
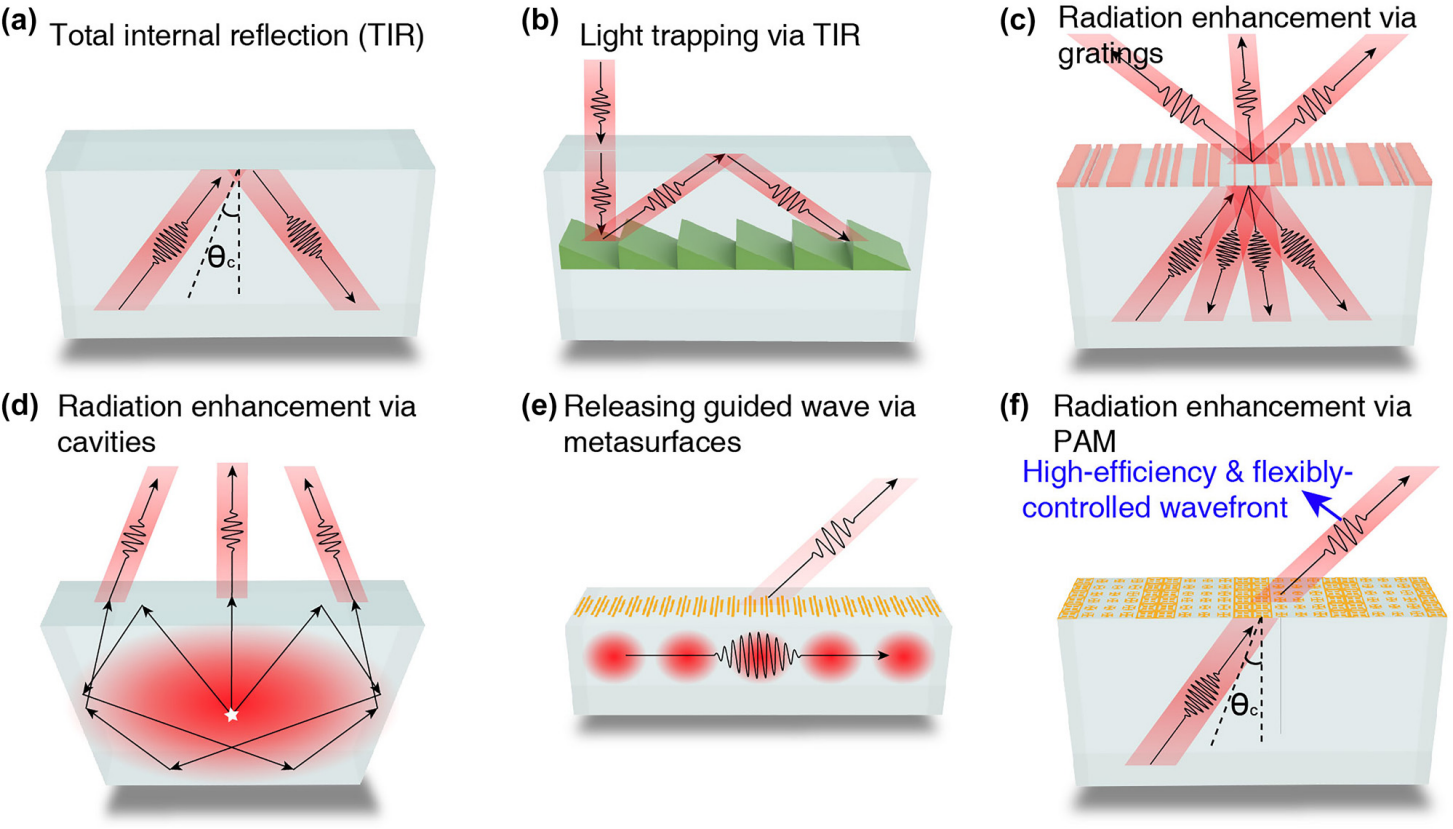
Schematic and application of TIR and several methods breaking through TIR. (a) Schematic diagram of TIR. (b) TIR is adopted to trap the sunlight in solar cells. (c) The grating structure leads to multiple transmitted and reflected beams under TIR conditions. (d) A resonant cavity can be used to increase the emitting efficiency of LEDs. (e) A metasurface on the waveguide can mold the guided waves into any desired free-space modes. (f) Schematic diagram of the proposed high-efficiency transmission with a flexibly-controlled wavefront beyond the critical angle via phase-gradient antireflection metasurfaces.

For electromagnetic waves incident from a higher-refractive-index medium to a lower-refractive-index one, there exists a critical angle beyond which all the incidence will be totally reflected, that is the so-called total internal reflection (TIR) [1], as schematically shown in Figure 1a. The TIR has been adopted in many electromagnetic devices due to its powerful wave confine functionality. A classic example is the dielectric waveguides [2] such as optical fibers, where the waveguide modes do not leak due to the TIR effect [2], [3], [4]. TIR also plays a crucial role in photovoltaics. Some solar cells utilize the TIR to harvest [5] and trap [6], [7] solar light to increase the absorption efficiency via blazed gratings [5], [6], as shown in Figure 1b, and phase-gradient metasurface [7]. However, on the other hand, it severely reduces the efficiency of radiation from a dielectric substrate such as the light-emitting diodes (LEDs) [8], [9]. In order to enhance the radiation beyond the critical angle, an additional lateral wave vector should be introduced on the interface [10]. One traditional method is to adopt the grating structure on the interface [9], [11], [12], as shown in Figure 1c, but it has two significant disadvantages. The first one is the relatively low transmission efficiency due to the presence of reflection [9], [11]. The second one is that the transmission is usually distributed among many diffraction orders [9], [11], thus significantly lowering the radiation efficiency along the desired particular angle. Resonant cavities [8] are also introduced to the LEDs to increase the emitting efficiency limited by the TIR. Radiation lights may experience multiple reflections on the reflective boundary and the radiation windows under the TIR condition and then emit from the radiation window with a small incident angle, as schematically shown in Figure 1d. However, the dispassion during multiple reflections lowers the total efficiency. In addition, the transmitted wavefront is fluctuated [8], resulting in low radiation efficiency along the desired particular angle.

Metasurfaces [10], [13], [14], [15], [16], [17] are planar artificial structures composed of arrays of subwavelength resonators that can generate flexibly-controlled phase-shift distributions on a surface and hence impose configurable wave vectors. Therefore, the metasurface is one of the most suitable candidates for enhancing radiation under the TIR condition. Actually, the pioneering work of phase-gradient metasurfaces [10] has reported that the critical angle of the TIR can be enlarged. However, the general existence of multiple beams of reflection and transmission hinders the efficiency, similar to the case of gratings shown in Figure 1c. Metasurfaces have also been applied on the surface of a dielectric waveguide to transform guided waves into deflected beams in the free space [18], as schematically shown in Figure 1e. However, under this circumstance, the radiation efficiency of each meta-atom is relatively low, such that the deflected beam exhibits a homogeneous intensity distribution. It is thus an interesting task to explore a mechanism for achieving high-efficiency radiation with a flexibly-controlled wavefront beyond the critical angle, which requires not only the compensation of wave vector, but also near-perfect impedance matching at large angles [19], [20], [21].

In this work, we demonstrate a metasurface-based solution for achieving high-efficiency transmission beyond the critical angle. As schematically shown in Figure 1f, incident waves at angles larger than the critical angle in dielectrics can perfectly transmit through the surface into free space by applying the so-called phase-gradient antireflection metasurfaces (PAM) [22], [23] on the surface. The PAMs are a unique class of metasurfaces featuring the combined functionalities of wave vector compensation and antireflection. They can function as the coalescence of metasurfaces and antireflection coatings in a deep subwavelength thickness. Previously, such a mechanism has led to the realization of “invisible” surfaces [22] and through-wall wireless communications [23], but those functions were still limited by the critical angle, due to the failure of traditional metasurface design strategy under the TIR condition. In order to circumvent the limitation of TIR, here we apply the reciprocity principle [24], [25], which has recently led to the discoveries of anomalous Brewster effect [26], [27] and transparent matte surfaces [28], [29], [30], in the metasurface design. As a result, a PAM working in microwave frequencies has been designed and experimentally fabricated, validating the phenomenon of high-efficiency radiation beyond the critical angle. Furthermore, we also demonstrate a PAM that can largely increase the total radiation from a point source embedded inside higher-refractive-index materials. Our work thus opens up a general and practical approach for achieving high-efficiency dielectric devices beyond traditional ones limited by the TIR.

## 2 Results and discussion

### 2.1 Reciprocity-based design strategy of PAMs working under the TIR condition

**Figure 2:**
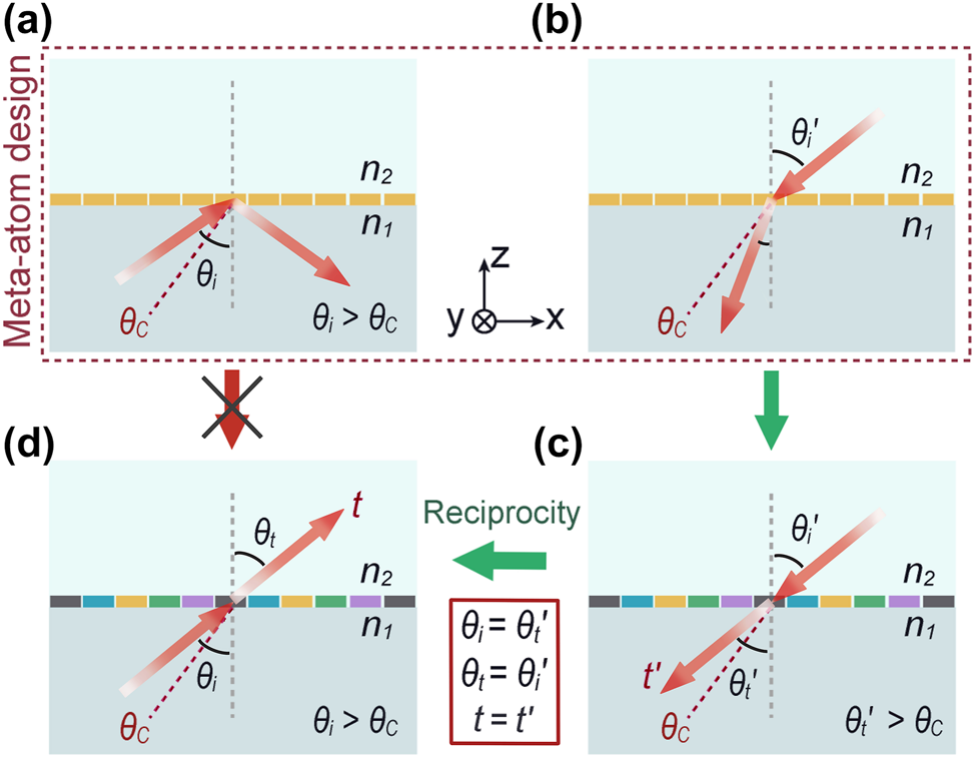
Design principle of the PAM operating under the TIR conditions. (a) The traditional strategy for the meta-atom design results in zero transmission under TIR conditions regardless of the specific structures and materials of the meta-atom. The red dashed line depicts the critical angle. (b) Meta-atom design for metasurface operating under incidence from the lower-refractive-index material is free from the TIR effect. (c) A PAM operates under incidence from the lower-refractive-index material. The anomalous deflection angle can exceed the critical angle, i.e., $\theta_{t}^{\prime }>\theta_{C}$ . (d) The PAM capable of high-efficiency transmission under the TIR conditions. Such a phenomenon is achieved by applying reciprocity operation to the beams in (c).

We consider a PAM on the interface between two dielectric materials with refractive index of *n*_1_ and *n*_2_ (*n*_2_ < *n*_1_). The purpose of this PAM is to achieve high-efficiency transmission (radiation) with a configurable angle of refraction, under incidence at incident angles larger than the critical angle, i.e., $\theta_{i}>\theta_{C}=Arcsin\left(n_{2}/n_{1}\right)$ , from the side of higher refractive index. The traditional strategy for designing phase-gradient metasurfaces first requires manipulating the transmission amplitude and phase shift of each meta-atom in a two-dimensional array. However, this approach cannot be directly applied to designing this high-efficiency PAM beyond the critical angle. This is because the design of each meta-atom is largely influenced by the asymmetric background with refractive index difference, and the transmittance is always zero under the incidence at the incident angle *θ*_*i*_ > *θ*_*C*_ from the side of higher refractive index *n*_1_ due to TIR, as shown in Figure 2a.

To circumvent this design difficulty, we apply the reciprocity principle. First, we consider incidence from the side of lower refractive index *n*_2_, as shown in Figure 2b. The traditional metasurface design principle is valid under this circumstance. The transmission coefficients of meta-atoms can be manipulated by engineering the structures and materials of meta-atoms based on various mechanisms like resonance-based manipulation [10], [13], [14], [22], [31], geometric phases [32], [33], and propagation phase accumulation [34], [35], etc. Then, a PAM can be constructed by arranging different meta-atoms with high transmittance and gradient transmission phase shift, so as to introduce the additional wave vector. When the additional wave vector is designed such that the lateral wave vector of the transmitted waves is enlarged, the angle of refraction can go beyond the critical angle. As shown in Figure 2c, under the incidence at an angle $\theta_{i}^{\prime }$ from the side of *n*_2_, this PAM can deflect the refractive beam to an angle of refraction $\theta_{t}^{\prime }>\theta_{C}$ with high efficiency. Interestingly, since the whole system is reciprocal, the reciprocity principle guarantees that when the incidence channel is switched with the transmission channel, the transmission coefficient is exactly the same, i.e., *t*′ = *t* [28], [29]. Hence, when a beam is incident from the side of *n*_1_ on the PAM at the angle of $\theta_{i}=\theta_{t}^{\prime }>\theta_{C}$ , the beam should be deflected to the angle of $\theta_{t}=\theta_{i}^{\prime }$ in the side of *n*_2_, as schematically shown in Figure 2d. Therefore, a PAM enabling high-efficiency radiation beyond the critical angle can be finally achieved. In the following, we demonstrate two practical PAMs applicable to incidence beyond the critical angle by adopting this reciprocity-based design strategy.

### 2.2 Experimental verification of high-efficiency anomalous refraction beyond the critical angle

**Figure 3:**
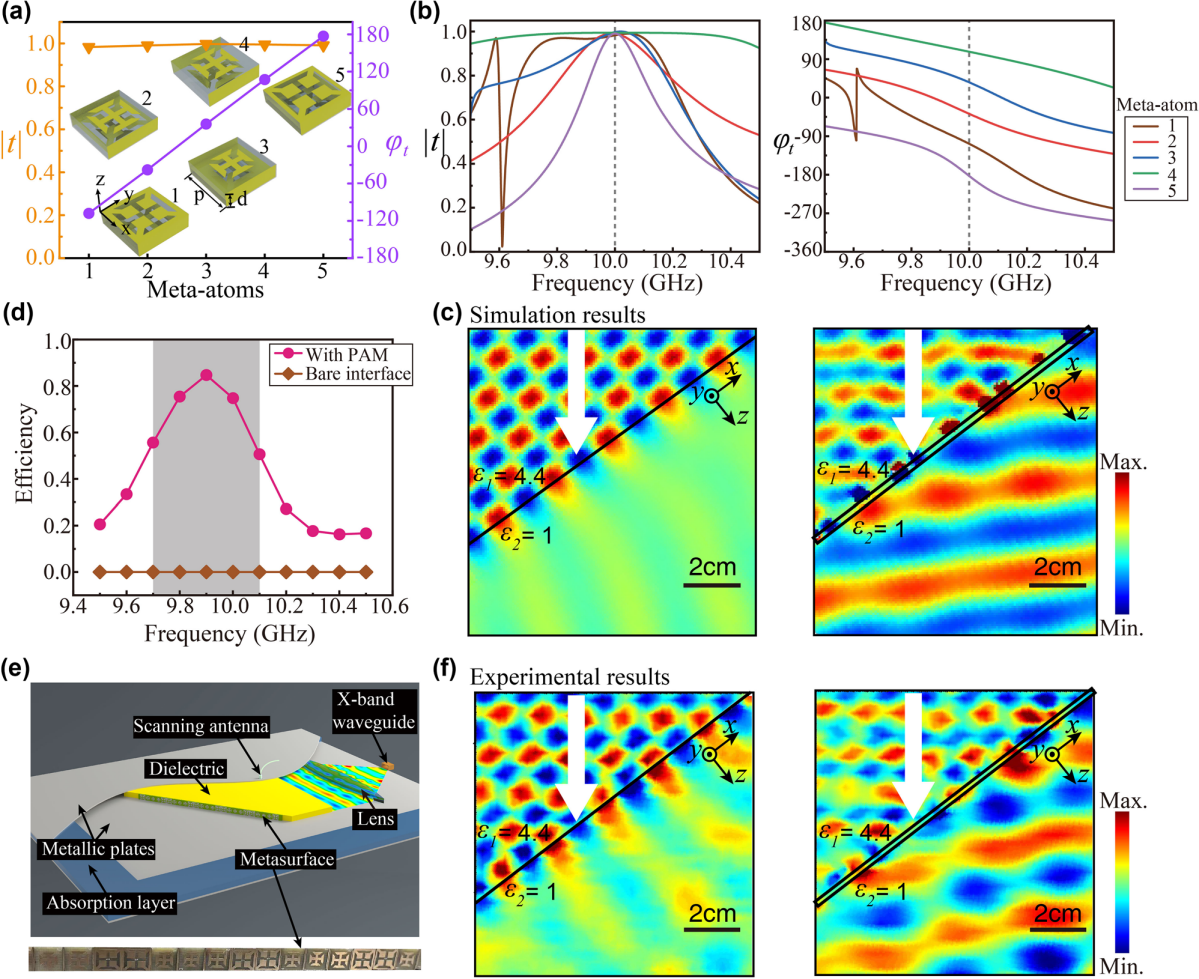
High-efficiency anomalous refraction beyond the critical angle. (a) The simulated transmission amplitude $\left|t\right|$ and phase shift *φ*_*t*_ of the five meta-atoms. These meta-atoms are set on the interface between two dielectric media with relative permittivity of 1 and 4.4, respectively. An oblique incidence with $\theta_{i}^{\prime }=30.0^{◦}$ from the lower permittivity side is assumed. (Inset) Three-dimensional diagram of these meta-atoms, where the period and thickness are *p* = 7.7 mm and *d* = 2 mm, respectively. (b) Spectra of the simulated $\left|t\right|$ and *φ*_*t*_ of the five meta-atoms. (c) The simulated electric field distributions of an oblique incidence at 10 GHz impinging on an interface without (the left panel) and with (the right panel) the designed PAM on it at an incident angle of *θ*_*i*_ = 37.5°. (d) Total transmission efficiencies of the PAM and a bare interface. (e) Schematic diagram of the experimental setup. The inset shows a photograph of the fabricated PAM. (f) The measured electric field distributions at 10 GHz.

In the first example, we design a practical microwave PAM that can generate high-efficiency anomalous refraction under incidence beyond the critical angle. We consider an interface between two dielectric materials with the relative permittivity of *ɛ*_1_ = 4.4 and *ɛ*_2_ = 1, and assume an incidence from the side of higher refractive index with *θ*_*i*_ = 37.5°, which is beyond the critical angle of $\theta_{C}=Arcsin\left(1/\sqrt{4.4}\right)\approx 28.5^{◦}$ , and a refraction angle of *θ*_*t*_ = 30.0°. Following the reciprocity-based design principle, the PAM should be designed under the assumption of $\theta_{i}^{\prime }=30.0^{◦}$ , $\theta_{t}^{\prime }=37.5^{◦}$ , and that the incidence is from the side of lower refractive index. According to the generalized Snell’s law [10]:

(1) $$n_{1}\sin\theta_{t}^{\prime }-n_{2}\sin\theta_{i}^{\prime }=\frac{\lambda }{2\pi }\frac{d\varphi }{dx},$$

where *λ* represents the wavelength in the vacuum, the phase gradient of the metasurface d*φ*/d*x* can be calculated. Considering a periodic supercell composed of 5 meta-atoms with a total phase shift of 360.0°, the phase shift difference between neighboring metaatoms is Δ*φ* = 72.0°. In addition, the transmittance of each meta-atom should be close to 1 to realize high-efficiency anomalous refraction. The working frequency is set as *f*_0_ = 10 GHz. Without loss of generality, the incidence is assumed as transverse electric (TE) polarized, i.e., the electric field is along *y* direction. We design five meta-atoms exhibiting high transmission and transmission phase difference near 72.0° by using structures of three-layer metallic patterns separated by two 1 mm-thick dielectric spacers with a relative permittivity of 4.4. Insets of Figure 3a show the three-dimensional diagram of these meta-atoms with a periodic of *p* = 7.7 mm and a thickness of *d* = 2 mm, which is only *λ*/15, i.e., in the deep subwavelength scale. Metallic sheets are used on the left and right sides of each meta-atom to suppress the mutual coupling between neighboring meta-atoms [22], [31]. It is worth noting that dielectric structures like silicon pillars [34], [35], [36] can also exhibit quite high transmission efficiency. Mutual coupling between neighboring silicon pillars is intrinsically weak since the waveguide modes are concentrated within the high-refractive-index materials. The detailed geometries of the metal patterns in the five meta-atoms are shown in Supplementary Figure 1. The transmission coefficients of these five meta-atoms at *f*_0_ are shown in Figure 3a, where the transmittance amplitudes are near one and the transmission phases are almost equally spaced, which are very close to the requirements from the theoretical analysis. The spectra of the transmission coefficient of these five meta-atoms near *f*_0_ are shown in Figure 3b, where the left and right panels show the transmission amplitude and phase, respectively.

By arranging the five meta-atoms sequentially and periodically, we construct a PAM operating on the dielectric interface that can result in high-efficiency anomalous refraction at *θ*_*t*_ = 30.0° under incidence at *θ*_*i*_ = 37.5°, which is beyond *θ*_*C*_. Numerical simulations based on the finite-difference time-domain method are performed for cases with and without the PAM. For a bare interface without the PAM, all the incidence is reflected and the transmission is negligible due to the TIR effect, as shown in the left panel of Figure 3c. However, when the PAM is applied, anomalous refraction is evident as shown in the right panel of Figure 3c, which is in sharp contrast to the case of bare interface, verifying the functionality of the PAM. The angle of refraction is very close to the theoretical prediction, i.e., *θ*_*t*_ = 30.0°. The slight wavefront fluctuations in the transmitted waves and the imperfect efficiency are primarily due to the discretization of the metasurface and the coupling between neighboring units, as previously observed in other metasurfaces [22], [37]. Figure 3d plots the total transmission efficiency spectrum of the PAM and a bare interface, which is calculated by integrating the power flow of the incident and refracted beams. The calculated transmission efficiency reaches 74.4 % at 10 GHz. A peak value of 84.7 % appears at the 9.9 GHz. The total transmission efficiency is over 50 % at frequencies ranging from 9.7 GHz to 10.1 GHz, which is defined as the operating band of the PAM. Contrarily, the total transmission efficiency of a bare interface is zero. We further verify this PAM via microwave experiments, where a row of meta-atoms of the PAM along *x* direction is fabricated and sandwiched by two parallel metallic plates with a distance of *p* = 7.7 mm to mimic a PAM periodically repeated in *y* direction, as schematically shown in Figure 3e. A photograph of the fabricated PAM is shown in the inset in Figure 3e. The experimental setup of this measurement is presented in detail in Ref. [22]. The measured results are plotted in Figure 3f for the case without (the left panel) and with (the right panel) the PAM, which are in good agreement with the simulated results. The slight discrepancy between the field distributions of the numerical and experimental results is mainly attributed to the fabrication error of the PAM and a tiny air gap between the metasurface and the upper metallic plate, which is equivalent to an additional gap in the *y* direction between each row of the PAM.

We have also investigated the incident-angle dependence of this PAM’s performance. The calculated transmission efficiency spectra of the PAM and a bare interface for comparison as a function of the incident angle are shown in Supplementary Figure 5. From Supplementary Figure 5, it can be seen that the transmission efficiency of the PAM is over 70 % over the incident angles range of 0°–40°, which indicates that the PAM is quite tolerant to the incidence angle, even though the PAM is designed for an incident angle of 37.5°. One may also find that the transmission efficiency of the PAM is significantly enhanced compared to the zero transmission of the bare interface at incident angles beyond the critical angle, verifying the functionality of the PAM. We note that the incident-angle tolerance of the PAM can be further extended by introducing precise angular-dispersion engineering [38], [39], [40].

It is worth noting that the reflection coefficients of a metasurface under incidences of TE and TM polarized oblique waves are different, especially for large incident angles. Therefore, the performances of this PAM under TE and TM polarized oblique incidence are different, though each meta-atom of the metasurface is isotropic. We investigate the performance of this PAM under a TM-polarized oblique incidence by numerical simulations. The calculated total transmission-efficiency spectrum of this metasurface is shown in Supplementary Figure 3. It is seen that the transmission efficiency peak under TM polarization is shifted to 9.8 GHz and the peak value is decreased to 78 %. The simulated magnetic field (y-component) distributions of the PAM under a TM-polarized oblique incidence at 9.8 GHz are in Supplementary Figure 4, where passable antireflection and wave deflection functionalities are observed. We note that the acceptable performance of the PAM under TM polarization should be attributed to the small incident angle of $\theta_{i}^{\prime }=30.0^{◦}$ .

### 2.3 PAM-enabled high-efficiency radiation of a point source embedded in dielectrics

**Figure 4:**
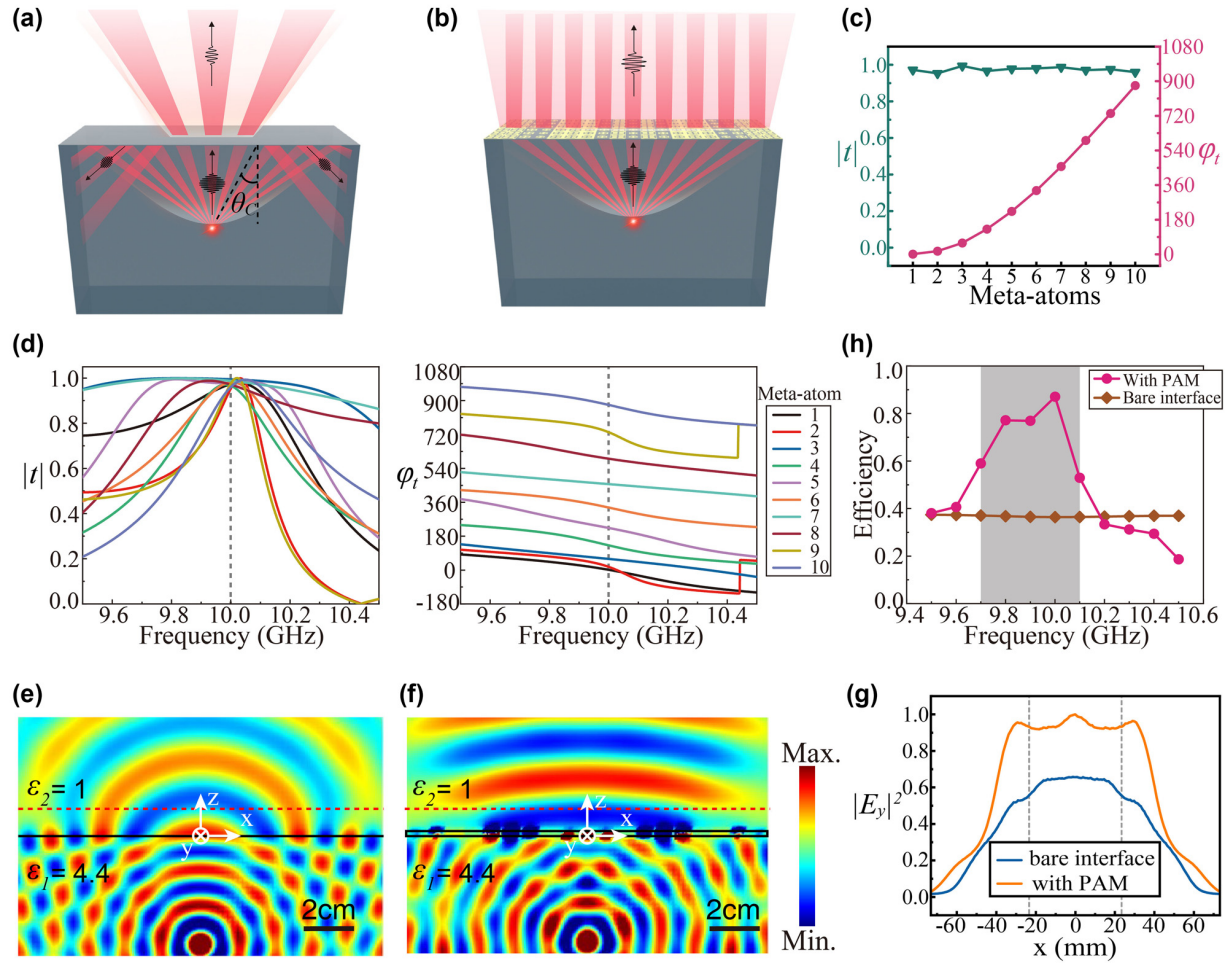
High-efficiency radiation of a point source enabled by a PAM. (a) The radiation efficiency of a point source in a dielectric block is limited due to the impendence mismatch and the TIR effect. (b) The radiation efficiency can be increased significantly by covering a PAM on the interface. (c) The simulated transmission amplitude $\left|t\right|$ and phase shift *φ*_*t*_ of meta-atoms. These meta-atoms are set on the interface between two dielectric media with relative permittivity of 1 and 4.4, respectively. A normal incidence from the lower permittivity side is assumed. (d) Spectra of the simulated transmission amplitude $\left|t\right|$ and phase shift *φ*_*t*_ of the five meta-atoms. (e, f) The simulated electric field distributions of a point source in a dielectric material with a refractive index of *ɛ*_1_ = 4.4 without (d) and with (e) the designed PAM on the interface. (g) The normalized electromagnetic wave intensity ( ${\left|E_{y}\right|}^{2}$ ) distributions along the *x* direction in the free space as depicted by the red dashed line in (d) and (e). The gray dashed lines represent the *x* coordinate corresponding to *θ*_*C*_ on the interface. (h) Total transmission efficiencies of the PAM and a bare interface under a TE-polarized normal incidence.

In the second example, we consider the radiation from a point source embedded in a dielectric block into free space. Waves impinging on the surface of the dielectric block at an incident angle *θ*_*i*_ < *θ*_*C*_ are partially reflected, while those at *θ*_*i*_ > *θ*_*C*_ are totally reflected, as illustrated schematically in Figure 4a. Moreover, waves radiated into the free space are diverged when propagate through the interface, similar to the effect of a concave lens. These are critical factors limiting the light-extraction efficiency of light-emitting diodes [8], [41] and quantum dots [41], [42], [43], where the light sources are embedded in a dielectric material. We note that dressing a PAM on this interface can address these issues. Transmission efficiencies of incidences with both *θ*_*i*_ < *θ*_*C*_ and *θ*_*i*_ > *θ*_*C*_ can be increased due to the impendence matching and the transverse wave vectors matching effects, as schematically shown in Figure 4b. What’s more, the transmitted wave can be manipulated to the desired direction by engineering the phase shift distributions of the PAM.

For a PAM that can highly efficiently convert radiation from a point source to normal transmission, its phase shift distributions can be expressed as [34], [35], [44]

(2) $$\varphi \left(x\right)=-\frac{2\pi n_{i}\sqrt{d^{2}+x^{2}}}{\lambda },$$

where *x* is the coordinate on the PAM and *d* = 3*λ*/*n*_*i*_ represents the distance from the source to the interface. We assume a PAM composed of 19 meta-atoms with a side length of *p*. The center of the PAM is right above the point source and is set as *x* = 0. Due to the symmetry of the metasurface along *x* direction, 10 meta-atoms with high transmittance and various phase shifts conform to Eq. (2) are required. Adopting the proposed design strategy, we design 10 meta-atoms with structures similar to that in Figure 3a. The detailed geometries of the metal patterns in the 10 meta-atoms are shown in Supplementary Figure 2. The transmission coefficients of these 10 meta-atoms at *f*_0_ are plotted in Figure 4c, where the transmission amplitudes are near unity and the corresponding transmission phases are in accord with Eq. (2). The spectra of the transmission coefficient of these 10 meta-atoms near *f*_0_ are shown in Figure 4d, where the left and right panels show the transmission amplitude and phase, respectively. A PAM can then be constructed by using these meta-atoms. Again, numerical simulations are performed for cases with and without the PAM. For a bare interface without the PAM, incidence from the point source is reflected and the transmitted wave is diverged, as shown in Figure 4e. Contrarily, when the PAM is covered on the interface, the transmitted wave is enhanced and propagates to the direction roughly normal to the interface, as shown in Figure 4f. To quantitatively study the performance of the PAM, Figure 4g plots the normalized wave intensity proportional to ${\left|E_{y}\right|}^{2}$ along *z* = 10 mm, which is depicted by red dashed lines in Figure 4e and f. It is found that the transmitted wave is significantly increased, demonstrating the effectiveness of the PAM. Figure 4h plots the total transmission efficiency spectrum of the PAM and a bare interface, which is calculated by integrating the power flow of the incident from a point source and the transmission beams. The calculated transmission efficiency of the PAM reaches a peak value of 87.0 % at 10 GHz. The total transmission efficiency is over 50 % at frequencies ranging from 9.7 GHz to 10.1 GHz, which is defined as the operating band of the PAM. Contrarily, the total transmission efficiency of a bare interface is only around 36 %.

Despite such a significant transmission enhancement, one can also find that the metasurfaces’ performance is imperfect. Firstly, there are considerable reflections at the boundary of the PAM. These reflections are caused by the large deflection angle between the oblique incidence and normal transmission, which requires not only non-uniform transmittances of the meta-atoms but also fast phase-shift changes along the interface [45]. Therefore, these reflections might be reduced by introducing non-uniform transmittance via active meta-atoms or bi-anisotropic meta-atoms and by increasing the space sampling with smaller meta-atoms. Secondly, the wavefront exhibits a concave shape instead of the ideal shape of a plane wave as expected. This can be attributed to that the PAM can only focus the plane wave to a region instead of a pre-defined focal point due to the diffraction-limited focusing effect. A flatter wave front might be achieved by finely optimizing the location of the point source.

## 3 Conclusions

In this work, we propose and experimentally demonstrate a PAM-based solution for achieving high-efficiency transmission beyond the critical angle. This novel phenomenon is made possible by utilizing a reciprocity-based design principle to circumvent the limitation of TIR, such that both impendence matching and wave vector compensation are considered in the design of PAMs. By precisely engineering the phase shift distributions of the PAM, the wavefront of the transmitted waves can be flexibly manipulated, such as generating a planar wavefront corresponding to directive propagation, surpassing the limitations of traditional grating-based methods. This design principle, validated by both numerical simulations and proof-of-concept experiments, offers a promising path for developing high-efficiency radiation from dielectrics beyond the critical angle. Compared with previous works of PAMs for small angles limited within the critical angle [22], [23], our work breaks the bottleneck of TIR, and opens a practical route towards unprecedented dielectric devices with omnidirectional high-efficiency [20], [46], [47]. Such reciprocity-enabled designs [26], [27], [28], [29], [30] may inspire future innovations in advanced metasurface technologies.

## Supplementary Material

Supplementary Material Details
