# Supplementary material for: High-efficiency radiation beyond the critical angle via phase-gradient antireflection metasurfaces
Source: Nanophotonics. 2025 Feb 3;14(3):305–13. doi: 10.1515/nanoph-2024-0545 (PMC11831388; doi:10.1515/nanoph-2024-0545)
Supplement: Supplementary file 1 — Supplementary Material Details [file j_nanoph-2024-0545_suppl_001.docx]

Supplementary materials for

High-efficiency radiation beyond the critical angle via phase-gradient antireflection metasurfaces

Xiaoxuan Ma^1†^, Hainan He^1†^, Runqi Jia^1^, Hongchen Chu^2,*^, and Yun Lai^1,*^

^1^National Laboratory of Solid State Microstructures, School of Physics, and Collaborative Innovation Center of Advanced Microstructures, Nanjing University, Nanjing, 210093, China

^2^School of Physics and Technology, Nanjing Normal University, Nanjing 210023, China.

^†^ These authors contributed equally to this work.

[*chuhongchen@njnu.edu.cn](mailto:*chuhongchen@njnu.edu.cn) (H.C.); [laiyun@nju.edu.cn](mailto:laiyun@nju.edu.cn) (Y.L.)


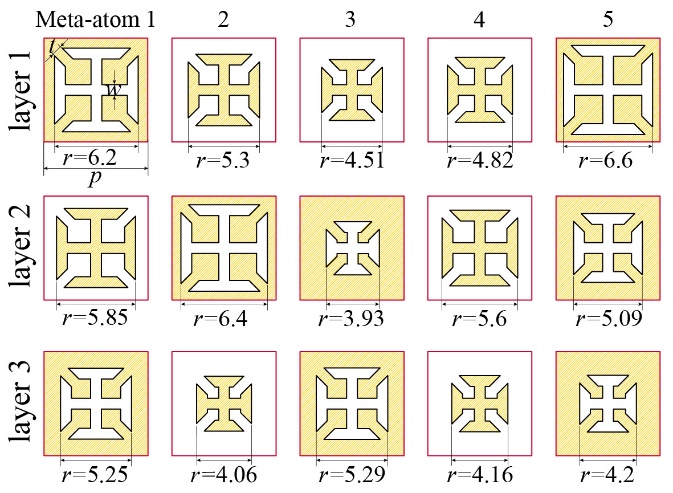


**Supplementary Figure 1.** Detailed geometries of the five meta-atoms of the GAM in Fig. 3. The shadow areas depict copper. The width of metal lines is $w=0.8 mm$. The out dimension, gap, and periodicity of the fourfold symmetric split-ring resonators are separately $r$, $t=0.8 mm$, and $p=7.7 mm$. The unit of these numbers of $r$ listed is $mm$.


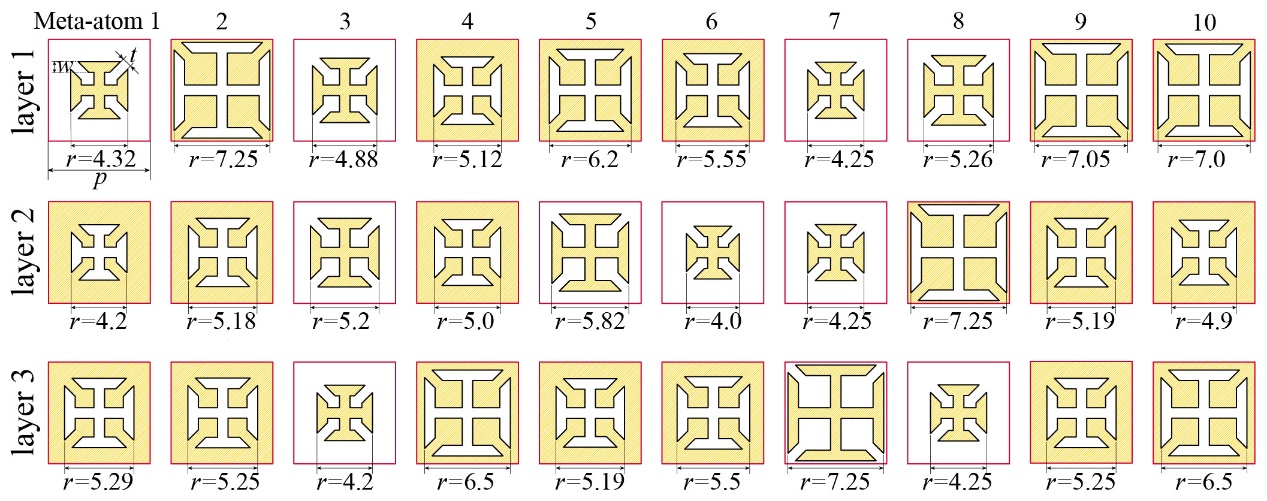


**Supplementary Figure 2.** Detailed geometries of the five meta-atoms of the GAM in Fig. 4. The shadow areas depict copper. The unit of these numbers listed in the insets is mm. The width of metal lines is $w=0.8 mm$. The out dimension, gap, and periodicity of the fourfold symmetric split-ring resonators are separately $r$, $t=0.8 mm$, and $p=7.7 mm$. The unit of these numbers of $r$ listed is $mm$.


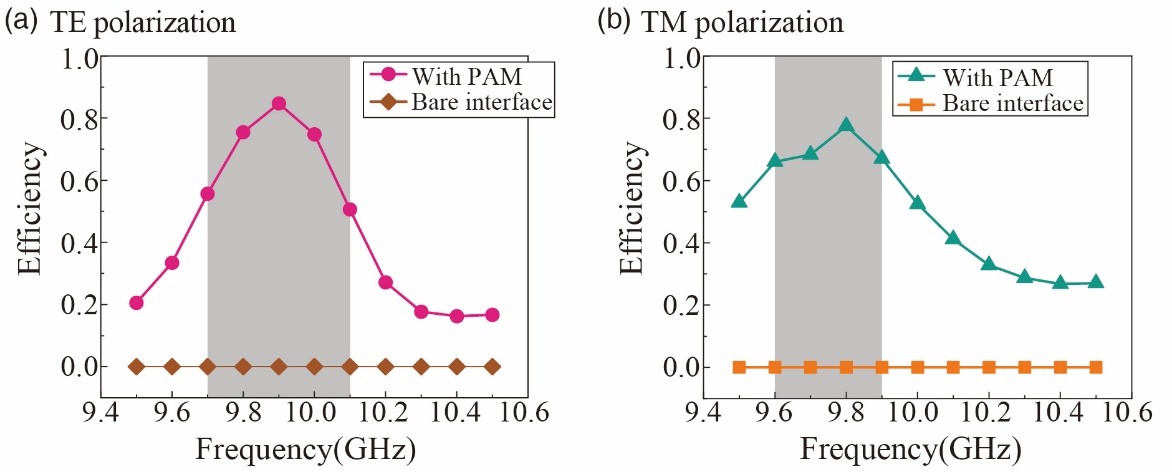


**Supplementary Figure 3**. Transmission efficiency spectra of the PAM in case 1 under TE and TM polarizations. The shadow regions depict a total transmission efficiency of over 50%.


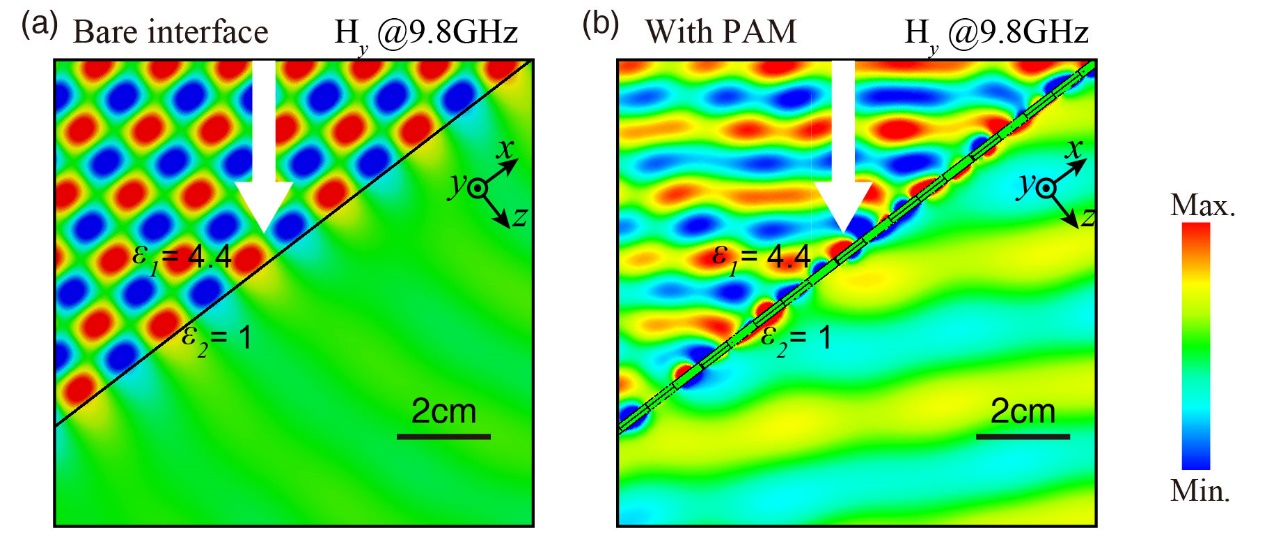


**Supplementary Figure 4**. The simulated magnetic field (y-component) distributions of a TM-polarized oblique incidence impinging on an interface without (the left panel) and with (the right panel) the designed PAM on it at an incident angle of $\theta_{i}=37.5^{\circ}$**.**


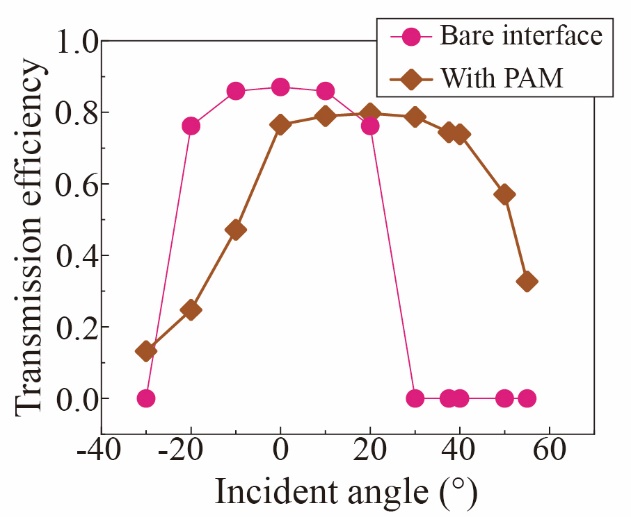


**Supplementary Figure 5**. Transmission efficiencies of the PAM in Fig. 3 and a bare interface as a function of incident angle.
